# Classification and automatic scoring of arousal intensity during sleep stages using machine learning

**DOI:** 10.1038/s41598-023-50653-9

**Published:** 2024-03-12

**Authors:** Hyewon Han, Min Jae Seong, Janghun Hyeon, Eunyeon Joo, Junhyoung Oh

**Affiliations:** 1 Department of Computer Engineering, Hongik University, Seoul, 04066 Republic of Korea; 2 Department of Neurology, Bundang Jesaeng Hospital, Seongnam, 13590 Korea; 3 Department of Artificial Intelligence Software, Hanbat National University, Daejeon, 34158 Republic of Korea; 4 Department of Neurology, Neuroscience center, Samsung Medical Center, Sungkyunkwan University School of Medicine, Seoul, 06351 South Korea; 5 Department of Digital Health, SAIHST, Sungkyunkwan University, Seoul, 06355 South Korea; 6 Division of Information Security, Seoul Women’s University, Seoul, 01797 Republic of Korea

**Keywords:** Data processing, Sleep

## Abstract

Arousal during sleep can result in sleep fragmentation and various physiological effects, impairing cognitive function and raising blood pressure and heart rate. However, the current definition of arousal has limitations in assessing both amplitude and duration, making it challenging to measure sleep fragmentation accurately. Moreover, there is inconsistency among inter-raters in arousal scoring, which renders it susceptible to subjective variability. Therefore, this study aims to identify a highly accurate classifier for each sleep stage by employing optimized feature selection and machine learning models. According to electroencephalography (EEG) signals during the arousal phase, the intensity level was categorized into four levels. For control, the non-arousal cases were used as level 0 and referred as sham arousal, resulting in five arousal intensity levels. Wavelet transform was applied to analyze sleep arousal to extract features from EEG. Based on these features, we classified arousal intensity levels through machine learning algorithms. Due to the different characteristics of EEG in each sleep stage, the classification model was optimized for the four sleep stages. Excluding sham arousals, a total of 13,532 arousal events were used. The lowest intensity in the entire data, level 1, was computed to be 3107, level 2 was 3384, level 3 was 3472, and the highest intensity of level 4 was 3,569. The optimized classification model for each sleep stage achieved an average sensitivity of 82.68%, specificity of 95.68%, and AUROC of 96.30%. The sensitivity of the control, arousal intensity level 0, was 83.07%, a 1.25% increase over the unoptimized model and a 14.22% increase over previous research. This study used machine learning techniques to develop classifiers for each sleep stage, improving the accuracy of arousal intensity classification. The classifiers showed high sensitivity and specificity and revealed the unique characteristics of arousal intensity during different sleep stages. These findings represent a novel approach to arousal research and have implications for developing more accurate predictive models in sleep research.

## Introduction

Various sleep disorders are closely associated with disruptions in standard sleep patterns, characterized by increased wakefulness or arousal. Arousal can result in sleep fragmentation, leading to a decline in cognitive function and autonomic reflex activation, increasing blood pressure and heart rate^1–4^. The standard definition of arousal is an abrupt shift in electroencephalogram (EEG) to a higher frequency, including alpha, theta, or beta, for at least 3 seconds, with at least 10 s of stable sleep preceding the change^5^.

Respiratory arousals during sleep are a significant predictor of the severity of obstructive sleep apnea (OSA) on polysomnography (PSG). While the severity of OSA is determined by the apnea-hypopnea index (AHI), there is evidence that the microstructure and intensity of arousal are closely correlated with the severity and could be the trait of the disease^6^. Despite the widespread use of the arousal index, which is defined as the frequency of arousals in PSG, to measure sleep fragmentation persists limited, as the standard definition from the American Academy of Sleep Medicine fails to consider variability in the duration and amplitude of EEG changes that meet this definition^7–9^. Additionally, discrepancies in arousal between scorers during the visual scoring process of PSG are still prevalent. In order to address these issues, researchers have begun to focus on the microstructure of arousal and have introduced the concept of arousal intensity^7^. Since research in this area is still in the emergent stage, there is currently no established definition of arousal intensity among researchers, leading to differences in the methodology used in each study.

In previous studies by Azarbarzin et al., a scorer visually scaled arousal intensity for each of the two EEG channels on a scale of 0 to 9, based entirely on EEG appearance within the most intense region of the arousal duration^8,9^. The study used a wavelet transform to extract specific features from the EEG signal related to arousal intensity to overcome the subjectivity and variability of visual scaling. The researchers trained seven different classifiers using different algorithms and values of k for k-nearest neighbor classifiers, with the training set made up of wavelet features and corresponding visually assigned arousal intensity scales. The study also found significant variability in arousal intensity among subjects, with increased heart rate response to arousal most strongly related to arousal intensity. This study introduced a method for automating the measurement of arousal intensity, which provides a tool to investigate the clinical consequences of arousal. A study by Amatoury et al. found that arousal intensity is a distinct pathophysiologic characteristic in OSA using this intensity scoring method^10^. In a recent study by Bahr et al., arousal intensity was defined as the amplitude of the electroencephalogram during respiratory arousals^11^. The distance between the highest and lowest point on the vertical axis of the EEG was defined as the amplitude of each respiratory arousal. The results showed strong positive correlations between the average arousal intensity and the respiratory disturbance and arousal indexes. All of the above studies are the results of efforts to overcome the limitations of the arousal index, such as inter-rater variability and the somewhat ambiguous and imprecise arousal definition.

Compared to the rapidly developing field of machine learning and various EEG analysis techniques, arousal analysis using these techniques is lagging. To the best of our knowledge, no studies have applied different updated machine learning techniques to analyze arousals and improve accuracy. Machine learning has shown remarkable applicability and potential in healthcare due to its ability and robustness to process high-dimensional data, resulting in recent inflation in research applying machine learning to various medical fields. EEG analysis is no exception to this trend, with diverse research exploring the application of machine learning^12^. Therfore, this study aims to utilize the concept of arousal intensity and apply it through machine learning techniques in order to address the limitations of traditional methods of measuring arousal.

In addition to medication and the presence of OSA, the sleep stage is also a significant factor affecting arousal^13^. Several studies have reported that arousal exhibits different characteristics depending on the sleep stage^11,14,15^. However, the relationship between sleep stage and arousal, in terms of arousal intensity and automated scoring, remains unknown. Recognizing the significant impact different sleep stages have on arousal, our study explores the void in the investigations conducted so far in understanding the relationship between sleep stage and arousal in terms of arousal intensity and automated scoring. Precisely identifying the unique characteristics of arousal intensity during different sleep stages could establish a more reliable measure of sleep fragmentation, which can help diagnose and treat sleep disorders by providing insight into the differences in arousal characteristics associated with various sleep disorders.

Our research aims to advance sleep research by introducing a new way to measure sleep arousal intensity. Automatically measuring sleep arousal intensity through applying machine learning and introducing increased precision through analyzing arousal intensity by each sleep stage will provide insights for diagnosing and treating sleep disorders.

**Figure 1:**
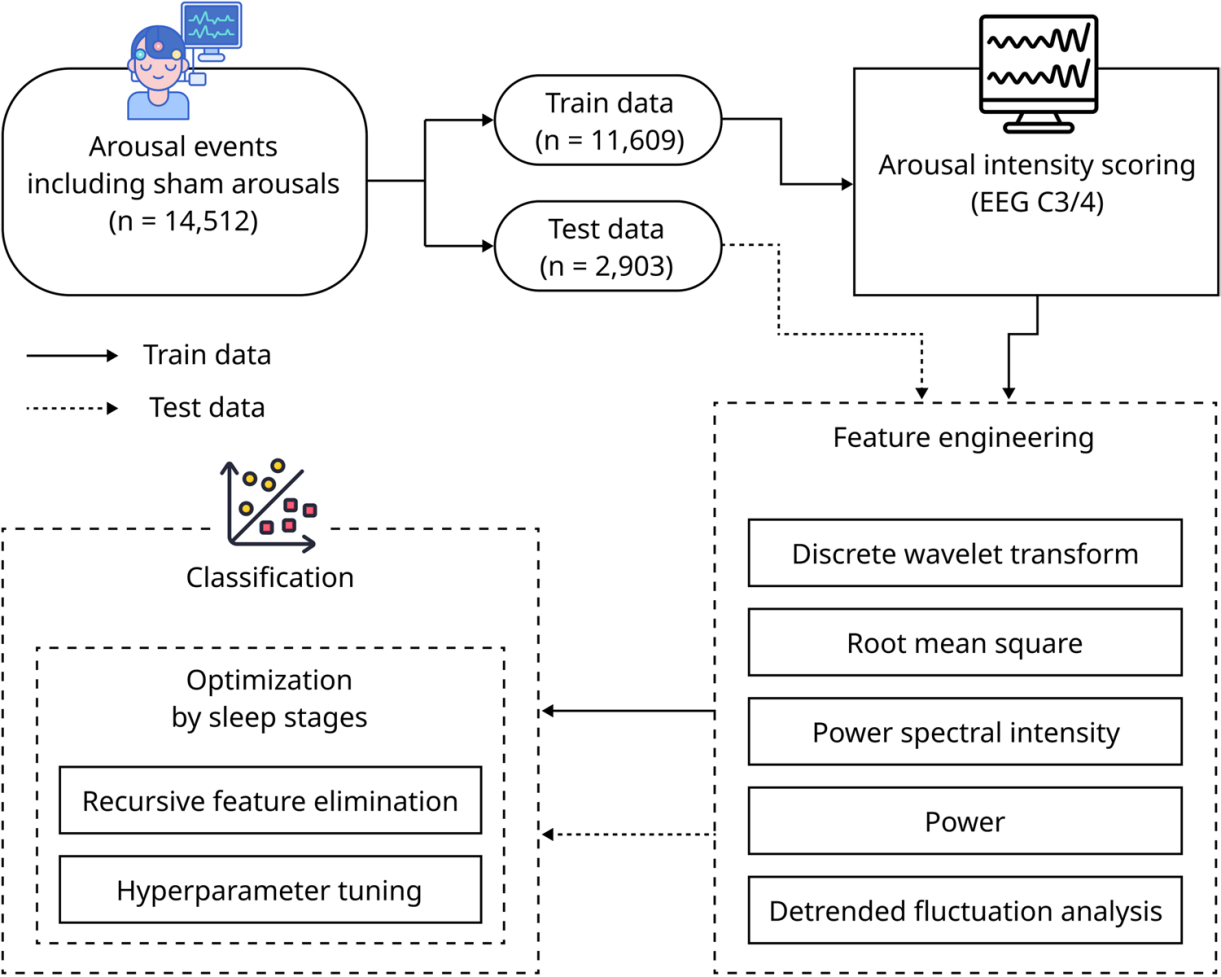
A workflow of the automated arousal intensity scoring and classification process. First, the collected arousal events are divided into training and test sets. The arousal intensity of the training data set is scored. Both datasets undergo a feature engineering process. Finally, the arousal intensity is classified using machine learning algorithms with sleep stage optimization. All icons from Freepik (freepik.com).

## Methods

### Data acquisition and ethical statement

Ethical approval was secured from the Institutional Review Board (IRB) at a local hospital (IRB no. 2023-02-078), ensuring the study’s conduct in a secure and ethical manner, with all data being anonymized. The IRB meticulously reviewed the study protocol and informed consent procedures. The study initially involved 101 patients diagnosed with OSA who underwent PSG between January and May 2022. The collected data encompassed PSG, EEG, and electromyography (EMG) features, along with participants’ demographics and sleep quality, as assessed through a structured questionnaire.

The analysis was confined to data interpreted by a single chosen scorer from multiple readers to curtail variability arising from potential scoring differences between scorers. Five of the 101 PSG results were deemed unusable due to the unavailability of EEG signal data, arousal event, and sleep stage data, culminating in a total of 96 valid results. A cumulative of 13,532 arousals were extracted from these 96 PSG results. A visualized workflow of the scoring and classification processes is delineated in Fig. 1.

The study utilized anonymized data and was conducted in compliance with the guidelines of the Declaration of Helsinki. Ethical approval was received from the Ethics Committee of Samsung Medical Center, Sungkyunkwan University (Ethical approval number: 2023-02-078), which carefully reviewed the study protocol and informed consent procedures to ensure the study was conducted in a safe and ethical manner. Informed consent was obtained from all study participants and their legal guardians.

### Automatic arousal intensity scoring

The previous study, which introduced the term “arousal intensity,” scored the intensity based on visual assessments of signal graphs of EEG channel C3/4 by an expert^8^. However, in the later research, arousal intensity was defined as the mean distance between the highest and lowest points of the amplitude from the C3 and C4 channels of the EEG signal^11^. Our study adapted the scoring method from the later study because it allows us to evaluate signals in a manner that most approximates the visual evaluation process while providing a more objective metric. Computed average distances were then binned into four levels of 1 to 4 using quantile. The intervals of the EEG signal and average distance for each level used for binning are shown in Table 1. To score intensity by contrasting stable sleep and arousal, 10 to 14 stable sleep periods of 9 seconds were selected from each PSG result. A total of 980 periods were selected, and we termed those “sham arousal,” as the previous research^8^. The intensity level was set as 0 since sham arousal is a period of stable sleep and not arousal. Therefore, five arousal intensity levels from 0 to 4 were constructed.

**Table 1:** Summarization of quartile range and intervals of the EEG signal used for scoring arousal intensity level.

Arousal intensity level	1	2	3	4
Quartile range	Q0–Q1 (0–25%)	Q1–Q2 (25–50%)	Q2–Q3 (50–75%)	Q3–Q4 (75–100%)
Interval of the EEG signal (V)	(-0.001, 0.000093]	(0.000093, 0.000164]	(0.000164, 0.000404]	(0.000404, 0.00603]

An arousal intensity level of 0 indicates that the event is not arousal, therefore, it is not used for quartile.

### Classification

#### Feature extraction

All data were processed to learn and classify the arousal intensity levels generated. The data used were EEG (C3/4, O1/2, F3/4), EMG (Chin), sleep stages, and arousal durations. The overall methods and procedures for signal processing and feature extraction were referred to in the previous work^8^. Discrete wavelet transform (DWT) is a signal processing method that decomposes a given signal into sets, where each set is coefficients and a pre-defined function called wavelet. Unlike the Fourier transform, which represents a signal in the frequency domain, the DWT represents a signal in both the time and frequency domains simultaneously; therefore, it is known as more suitable for processing EEG signals^16^. There are several types of these functions, and in this study, the Daubechies order 4 wavelet was used, which is considered the most suitable for analyzing EEG signals^17^.

**Figure 2:**
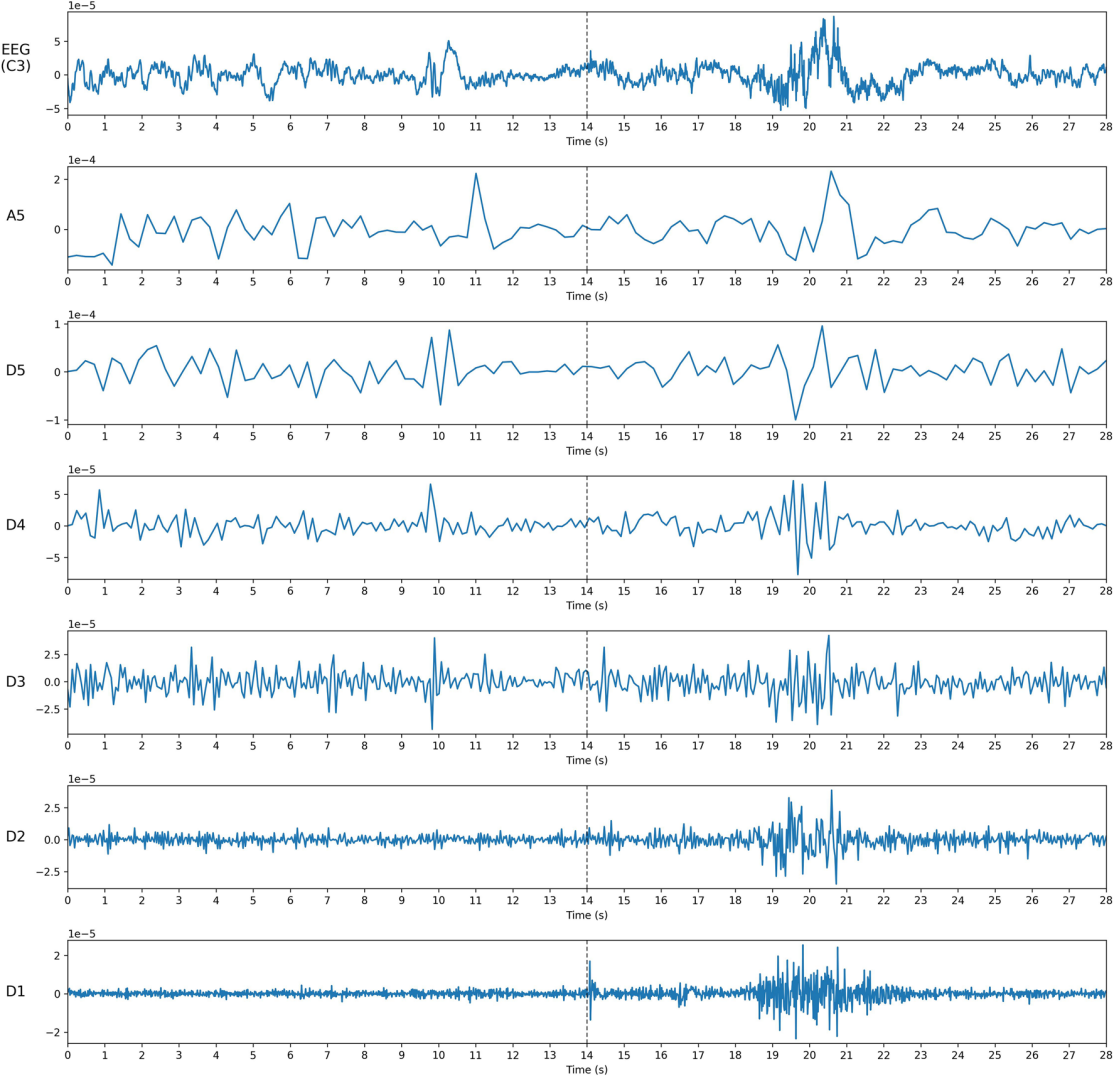
An example of EEG signal channel C3 before and after the arousal event. The dashed vertical line on the graphs indicates the onset of the arousal event. The first graph shows the raw signal, and the following visualizes coefficients of D_1_ to D_5_ and A_5_, respectively.

A 5-level DWT was performed on all signals from EEG and EMG for the arousal period and the same period before the arousal onset. Every preprocessing and analysis of the data was conducted with Python(version 3.9.9, Python Software Foundation), including the transformation. Before applying DWT, high-pass filtering with a cutoff frequency of 0.3 Hz was also applied to reduce the influence of the artifacts. 200 Hz EEG signals were resampled to 128 Hz for efficiency in signal processing. Figure 2 shows an example of the result of a 5-level DWT with a Daubechies order 4 wavelet on an EEG signal (C3). The calculated coefficients (D_1_-D_5_ and A_5_) were used to calculate the mean power, the mean of absolute values (MABS), the ratio of MABS for all combinations of each signal (e.g. $\frac{C3/4\; MABS(D_1)}{C3/4\; MABS(D_2)}, \frac{C3/4\; MABS(D_1)}{C3/4\; MABS(D_3)}, \frac{C3/4\; MABS(D_1)}{C3/4\; MABS(D_4)}, \frac{C3/4\; MABS(D_1)}{C3/4\; MABS(D_5)}, \frac{C3/4\; MABS(D_1)}{C3/4\; MABS(A_5)}$, ...), and the total variance. Thus, a total of 132 features were generated, and all those features were normalized by dividing the arousal value by the pre-arousal value.

In addition to the features computed using coefficients resulting from the DWT, four more feature extraction methods widely used in EEG signal analysis to obtain additional features were applied: average power spectral intensity (PSI)^18^, average power, average root mean square value (RMS)^19^, and average detrended fluctuation analysis (DFA)^20^. First, PSI provides information about the distribution of power across different frequencies. Second is average power, which provides the overall power of the EEG signal, irrespective of the frequency. Third, RMS shows information about the overall amplitude of the EEG signal. Lastly, DFA provides information about the complexity or irregularity of the EEG signal. Outcomes from the four techniques using the EEG C3/4 signals were used as additional features. As a result, a total of 136 features were generated.

Since there was not enough level 0 compared to other arousal intensity levels, a synthetic minority over-sampling technique (SMOTE) was applied to solve this data imbalance problem^21^. SMOTE is a technique that adopts the k-nearest neighbor algorithm to generate new data for underrepresented classes. It is one of the most common oversampling methods used to address the data imbalance problem in machine learning.

#### Classification and feature selection methods

Data were randomly selected and divided into a train set (80%) and a test set (20%). Two machine learning classifiers, random forest^22^ and LightGBM^23^, were used. Random forest is a classifier consisting of a combination of decision trees built on random sub-samples of the dataset. LightGBM is a gradient-boosting framework designed to be fast and highly efficient. Both are machine learning algorithms commonly used in various fields when applying machine learning techniques due to their outstanding performance.

To reduce the dimensionality of the data, we included the feature selection process, and recursive feature elimination (RFE)^24^ was used as the feature selection technique in this study. RFE is a prevailing feature selection method applied in machine learning. The method allows identifying and selecting the most important features in a dataset by recursively removing the least important ones. Cross-validation was employed in conjunction with RFE to enhance precision in this study. The feature selection procedure was performed for each classifier: random forest and LightGBM. The number of cross-validation was set to 5.

#### Optimization by sleep stages

Due to the different characteristics of each sleep stage, we optimized the classification model by sleep stages. Sleep can be divided into two broad categories: rapid eye movement (REM) sleep and non-REM (NREM) sleep. In REM sleep, diminished EMG tone is observed, and the EEG appears remarkably similar to the waking state with low-voltage, high-frequency^25^. NREM sleep can be further categorized into three stages: N1, N2, and N3.

In N1, EEG shows moderate amplitude and irregularly spaced bursts of slow waves. N2 is characterized by a further increase in EEG amplitude and the frequent appearance of sleep spindles with K-complexes. Sleep spindles are clusters of occasional high-frequency spikes that occur periodically and last for several seconds. K-complexes are defined as the occurrence of sharp, clear, high-voltage bipolar waves lasting more than half a second. N3 is also known as slow-wave sleep. A larger EEG amplitude also characterizes it compared to the previous stage. As the stage escalates, sleep becomes more profound, and muscle activity decreases.

**Table 2:** Descriptive Statistics of participants’ demographics and polysomnography.

Variables (n=96)	Mean ± SD
Age (years)	56.0 ± 14.6
Sex (male, %)	72 (75%)
Height (cm)	166.5 ± 9.1
Weight (kg)	73.5 ± 15.0
BMI	26.3 ± 4.0
PSQI	8.4 ± 4.0
ISI	12.3 ± 5.7
ESS	9.4 ± 4.7
BDI	12.6 ± 7.9
Polysomnography	
Total sleep time	317.6 ± 59.7
Sleep latency	13.7 ± 17.8
N2 latency	8.9 ± 17.0
REM latency	108.9 ± 63.8
WASO (%)	19.1 ± 10.8
NREM1/TST (%)	23.3 ± 14.7
NREM2/TST (%)	55.1 ± 12.8
NREM3/TST (%)	5.3 ± 5.9
REM/TST (%)	16.4 ± 6.8
Arousal index	32.3 ± 15.9
Respiratory	24.4 ± 18.3
Spontaneous	4.0 ± 6.2
Movement	0.7 ± 1.6
REM	29.5 ± 18.0
NREM	33.0 ± 16.7
Sleep efficiency (%)	78.3 ± 11.5
AHI	38.5 ± 23.4
RDI	40.9 ± 23.2
Lowest SaO2 (%)	82.3 ± 8.4
ODI (%)	32.1 ± 21.9
PLMS index	18.6 ± 24.1
MAI	0.7 ± 1.6

*BMI* body mass index,* PSQI* Pittsburgh sleep quality index,* ISI* insomnia severity index,* ESS* Epworth sleepiness scale,* BDI* beck depression inventory,* WASO* wake after sleep onset,* REM* rapid eye movement,* AHI* apnea-hypopnea index,* RDI* respiratory disturbance index,* SaO*_2_ oxygen saturation,* ODI* oxygen desaturation index,* PLMS* periodic limb movement in sleep,* MAI* movement arousal index.

During the classification process, we optimized performance for each of the four sleep stages: REM, N1, N2, and N3. The optimization was conducted through two phases: feature selection and hyperparameter tuning. For the hyperparameter tuning, a tree-structured parzen estimator (TPE) approach^26^, which is a method based on Bayesian optimization, was utilized to sample the hyperparameter space, and it was pruned with the hyperband algorithm^27^ for the efficiency of the process.

**Table 3:** Results of arousal intensity scoring using vertical distances of EEG signals (channel C3/4).

Arousal intensity level	Train set (n = 11,609)	Test set (n = 2903)	Total (n = 14,512)
0 (sham arousal)	782	198	980
1	2464	643	3107
2	2710	674	3384
3	2790	682	3472
4	2863	706	3569

The higher the arousal intensity level, the more intense the arousal. Since sham arousals indicate events that are not arousals, they are all assigned a level of 0. Therefore, an arousal intensity level of 0 means they are not an arousal.

## Results

### Demographic and polysomnographic characteristics of participants

Participants in the study had a mean age of 56.0 ± 14.6 years, and 72 (75%) were male. The polysomnographic data revealed a substantial arousal index at 32.3 ± 15.9, with the majority being respiratory at 24.4 ± 18.3. Notably, arousals were more prominent during non-REM (NREM) sleep, with an index of 33.0 ± 16.7 compared to 29.5 ± 18.0 during REM sleep.

The average AHI was marked at 38.5 ± 23.4, indicative of severe OSA. Correspondingly, the lowest saturation was noted at 82.3 ± 8.4%, and the oxygen desaturation index (ODI) was 32.1 ± 21.9, reflecting a significant desaturation burden attributable to OSA. The arousal index was 32.3 ± 15.9 with subcategories: respiratory 24.4 ± 18.3, spontaneous 4.0 ± 6.2, movement 0.7 ± 1.6, REM 29.5 ± 18.0, and NREM 33.0 ± 16.7. Sleep efficiency was 78.3 ± 11.5% (Table 2).

### Arousal intensity level scoring

A total of 13,532 arousal events, excluding sham arousals, were divided into four levels according to the proposed scoring approach. In the total data, the lowest intensity of level 1 was calculated as 3,107, level 2 as 3384, level 3 as 3472, and finally, the highest intensity of level 4 was computed as 3569. Table 3 summarizes the numbers of data for each arousal intensity level, including sham arousals, by the dataset.

**Table 4:** Determined number of features and classifiers for each sleep stage. Based on the trial result, LightGBM was used for REM stage and the random forest algorithm was used for all the other sleep stages.

Sleep stage	REM	N1	N2	N3
Classifier	LightGBM	Random forest	Random forest	Random forest
Number of selected features	31	31	36	11

Each number of selected features was also determined based on the trial results.

### Optimization

RFE with cross-validation was used to select features and determine the number of features. The feature selection process was conducted for each classifier and each sleep stage. Based on the average accuracy of 5 cross-validations, the combination of features with the highest mean accuracy was chosen. For the REM stage, the highest accuracy was achieved when 31 features were used with the LightGBM classifier. For the N1 and N2 stages, 31 and 36 features were selected, respectively, using the random forest. Lastly, for the N3 stage, 11 features were used using the random forest classifier. Table 4 summarizes the selected classifiers and features. Random forest classifiers were used in all the other stages except for the REM stage.

### Classification

**Figure 3:**
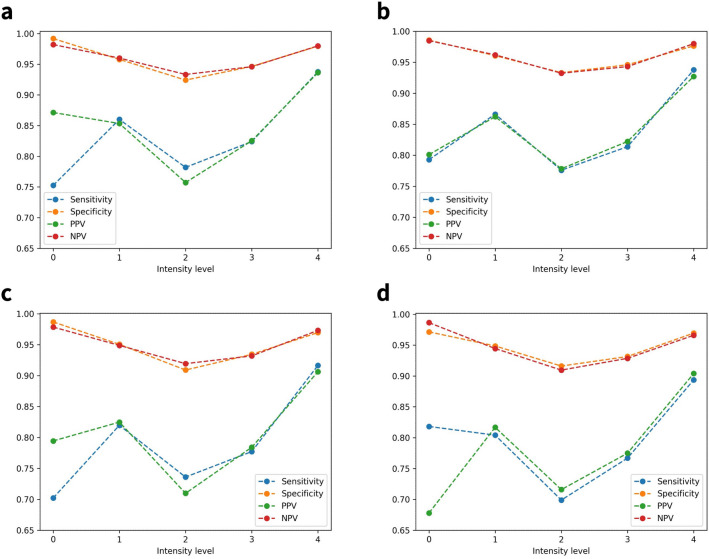
Sensitivity, specificity, negative predictive value (NPV), and positive predictive value (PPV) of arousal intensity level classification results without the optimization process for each sleep stage. Each machine learning algorithm was used with the default hyperparameter setting. (**a**) The metrics values of arousal intensity level classification results using a LightGBM classifier and SMOTE without the optimization process for each sleep stage. (**b**) The metrics values of arousal intensity level classification results using a LightGBM classifier and SMOTE without the optimization process for each sleep stage. (**c**) The metrics values of arousal intensity level classification results using a random forest classifier without the optimization process for each sleep stage. (**d**) The metrics values of arousal intensity level classification results using a random forest classifier and SMOTE without the optimization process for each sleep stage.

**Figure 4:**
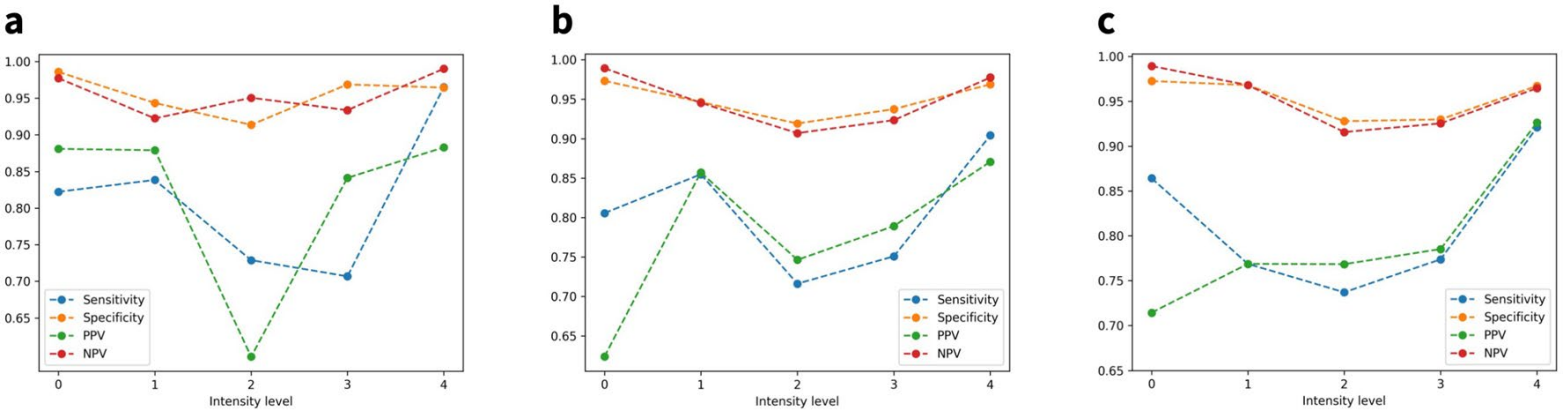
Sensitivity, specificity, negative predictive value (NPV), and positive predictive value (PPV) of arousal intensity level classification results for each sleep stage. Since all metric values are equal to the maximum value of 1.00 in the N3 stage, the graph of the stage was skipped for better representation. (**a**) The metrics values of the classification results for each arousal intensity level in the REM stage. (**b**) The metrics values of the classification results for each arousal intensity level in the N1 stage. (**c**) The metrics values of the classification results for each arousal intensity level in the N2 stage.

Sensitivity, specificity, negative predictive value (NPV), and positive predictive value (PPV) of the arousal intensity level classification results before optimization by sleep stage are shown in Fig. 3. Figure 4 visualizes the metrics of the results for each sleep stage. In the case of the N3 stage, the number of data belonging to this stage was relatively limited. Therefore, only level 3 and level 4 from the total five levels were present in the test set. The highest tier of arousal intensity, level 4, measured the highest scores for all evaluation metrics, regardless of the optimization. Without the optimization, all the outcome metrics for each intensity level in the classification results were comparable, but with the optimization, they differed marginally. This was especially noticeable in the results for REM sleep.

The optimization was driven by increasing the sensitivity of the control, sham arousal (i.e., arousal intensity level 0). Without the optimization process, the highest sensitivity of intensity level 0 using a single classifier was 81.82%, which was the case when using a random forest classifier and SMOTE simultaneously. When it optimized for each sleep stage, the average sensitivity of level 0 was 83.07%, an increase of 1.25% compared to the previous result. For the N2 stage, the sensitivity of arousal intensity level 0 was the highest among all the sleep stages, with a value of 86.42%.

**Figure 5:**
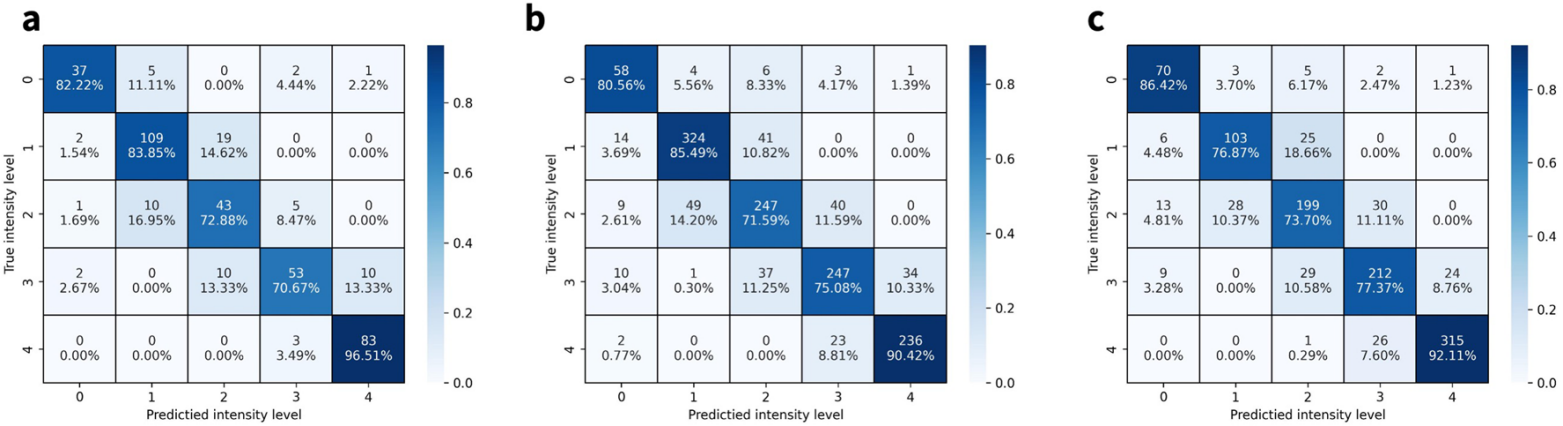
Confusion matrices of arousal intensity level classification results from each sleep stage. Since all metric values are equal to the maximum value of 1.00 in the N3 stage, the graph of the stage was skipped for better representation. (**a**) Confusion matrix of arousal intensity level classification results from the REM stage. (**b**) Confusion matrix of arousal intensity level classification results from the N1 stage. (**c**) Confusion matrix of arousal intensity level classification results from the N2 stage.

Confusion matrices of the results are shown in Fig. 5. While the classification is overall well achieved, there was an average of 14.70% of cases where level 1 was classified as level 2 and an average of 13.84% of cases where level 2 was classified as level 1. For an average of 10.39% of the arousals, level 2 was categorized as level 3, and an average of 11.72% of arousal intensity level 3 was allocated as intensity level 2. In the remaining levels, there were minor misclassifications for the continuous levels, but it was less than 10% in all cases.

**Figure 6:**
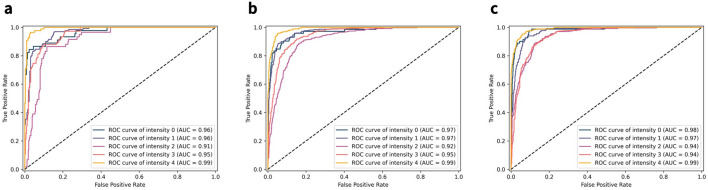
Receiver operating characteristic (ROC) curves of arousal intensity level classification results by sleep stages. Since all metric values are equal to the maximum value of 1.00 in the N3 stage, the graph of the stage was skipped for better representation. (**a**) ROC curves of arousal intensity level classification results from the REM stage. (**b**) ROC curves of arousal intensity level classification results from the N1 stage. (**c**) ROC curves of arousal intensity level classification results from the N2 stage.

The average AUC value for the receiver operating characteristic (ROC) curve for sham arousal was 97.07%. As for the sensitivity, the AUC for intensity level 0 was highest at stage N2, with a value of 97.95%. The ROC curves by arousal intensity level for all four sleep stages are illustrated in Fig. 6. All the performance metrics of the optimized classification model by sleep stages for the test set are summarized in Table 5.

### Relationship to prior works

While previous studies have primarily focused on using arousal intensity to investigate the relationship between sleep arousal and cardiovascular disease^8^ or to examine the association between sleep arousal severity and OSA^10^, this study took a different approach by focusing on developing an automated pipeline for the measurement and classification of arousal intensity. Unlike previous studies that relied heavily on manual assessment by experts to score arousal intensity, we introduced a fully automated pipeline, which not only simplified the process of scoring arousal intensity but also increased its potential for use in various studies.

While this research shares similarities with previous studies, particularly in its data processing techniques, we focused on designing and implementing an automated arousal intensity scoring algorithm. In particular, our study demonstrated that the classification algorithm outperformed previous studies. Since previous studies lacked a quantitative baseline, the only direct comparison in terms of performance is the classification result of sham arousal. In the previous study, 168 received the arousal intensity scale of 0, which was classified as sham arousal out of 244 sham arousal^8^. Therefore, the calculated hit rate of the previous study is 68.85%, and our proposed classifier achieved a hit rate of 83.07%, recording an improvement of 14.22% over previous results. This advancement represents an essential contribution to the field of sleep research and arousal intensity assessment.

**Table 5:** Summary table of sensitivity, specificity, negative predictive value (NPV), positive predictive value (PPV), and area under the receiver operating characteristic (AUROC) curves for arousal intensity classification results by the sleep stage-optimized model.

Sleep stage	Arousal intensity level	Sensitivity (%)	Specificity (%)	PPV (%)	NPV (%)	AUROC (%)	N
REM	0	82.22	98.57	88.10	97.73	96.44	45
	1	83.85	94.34	87.90	92.25	96.42	130
	2	72.88	91.37	59.72	95.05	91.08	59
	3	70.67	96.88	84.13	93.37	95.32	75
	4	96.51	96.44	88.30	99.00	99.32	86
N1	0	80.56	97.34	62.37	98.92	96.64	72
	1	85.49	94.64	85.71	94.54	97.03	379
	2	71.59	91.93	74.62	90.71	91.64	345
	3	75.08	93.76	78.91	92.36	94.72	329
	4	90.42	96.89	87.08	97.76	98.84	261
N2	0	86.42	97.25	71.43	98.90	97.95	81
	1	76.87	96.79	76.87	96.79	97.16	134
	2	73.70	92.78	76.83	91.57	94.19	270
	3	77.37	92.99	78.52	92.54	94.10	274
	4	92.11	96.71	92.65	96.45	98.87	342
N3	3	100.00	100.00	100.00	100.00	100.00	4
	4	100.00	100.00	100.00	100.00	100.00	17
Average	0	83.07	97.72	73.96	98.52	97.01	198
	1	82.07	95.26	83.49	94.53	96.87	643
	2	72.73	92.03	70.39	92.44	92.30	674
	3	80.78	95.90	85.39	94.57	96.03	682
	4	94.76	97.51	92.01	98.30	99.26	706
	Total	82.68	95.68	81.05	95.67	96.30	2903

## Discussion

Sleep disorders, such as OSA, can severely impact an individual’s health, making accurate identification and classification of arousal intensity during sleep essential. However, the existing method of scoring arousal intensity involves the visual reading of all EEG recordings, which not only requires a dedicated human expert to perform the scoring but also makes all scores solely dependent on the subjective assessment of the expert. Therefore, our study aimed to overcome these shortcomings by proposing an automated sleep intensity scoring methodology. In addition, previous studies have utilized traditional machine learning methods to identify arousal precisely; however, this approach needs more accuracy. Therefore, this study aimed to develop a more accurate arousal intensity classification model according to sleep stage.

The classifier was optimized using feature selection and cross-validation techniques for each sleep stage, and the classification performance was evaluated using various metrics such as sensitivity, specificity, negative predictive value (NPV), positive predictive value (PPV), and area under receiver operating characteristic (AUROC). The results indicated that the proposed model achieved high sensitivity and specificity, particularly for the highest intensity level 4. The optimization process improved the sensitivity of control and sham arousal (level 0), which was essential for minimizing false positives and improving the overall accuracy of the model. The potential benefits of combining feature selection with other advanced techniques, such as machine learning, highlight the importance of ongoing research to identify new and innovative ways to optimize the performance of machine learning models. Previous studies utilizing machine learning models such as naive Bayes, decision tree, support vector machine, generalized linear model, and k-nearest neighbor have reported an accuracy range of approximately 81% to 93% for predictive power and a sensitivity range of 75% to 83% for arousal detection^28–31^. The models utilized in this study included LightGBM and random forest, which have demonstrated effectiveness in various classification tasks. Therefore, the strength of the machine learning model used in our study lies in its ability to provide more precise classification and access to the arousal microstructure.

In addition, our study aimed to develop an accurate arousal intensity classifier based on sleep stage. The authors focus on the macroscopic aspects of arousal that are characteristic of arousal in OSA for each sleep stage, which provides an important foundation for our study. One researcher reported that the arousal threshold was found to be highest during N3 and lowest during N1 and N2, with REM having a lower threshold than N1 and N2^13^. These findings suggest that N3 sleep requires stronger stimuli to induce wakefulness and is less prone to progressing to wakefulness. Moreover, the arousal index is highest during N1 and lowest during N3, consistent with the arousal threshold^32^.

Although there are limited studies on the differences in the microstructure of arousal across sleep stages, a few studies have reported no significant difference in arousal intensity between healthy individuals and those with OSA, as well as between REM and NREM sleep^10^. However, it is difficult to conclude the relationship between arousal intensity and sleep stage based on visual scoring methods used in these studies, as appropriate classifiers for each sleep stage were not utilized.

Our study selected the combination of features with the highest mean accuracy for each sleep stage. The REM stage achieved the highest accuracy using 31 features with the LightGBM classifier, and the N1 stage selected 31 features with the random forest classifier. The N2 stage used 36 features with the random forest classifier, while the N3 stage used 11 features with the same classifier. Assuming that the microstructure of arousal is consistent across all sleep stages, it would be reasonable to expect that the selected feature and the classifier with the highest accuracy would demonstrate common characteristics. This represents a novel perspective in approaching arousal and its related factors and has important implications for developing more accurate and effective predictive models in sleep research.

However, there are some limitations to this study. Experiments could not be conducted using a more diversified set of machine learning models, and the optimization by sleep stages phase took a considerable amount of time since RFE, which selects features by removing features one by one and confirms the performance, was adopted. Another limitation is that since arousal intensity was calculated as the vertical distance from the EEG amplitude, time-frequency factors could not be included in the level calculation. In addition, we recognize that our data collection was limited to a local hospital, which may affect the diversity of participant composition. We also acknowledge that our study’s design, while robust, may not completely eliminate subject-related factors. In future research, exploring additional techniques for subject-independent analyses or conducting larger-scale studies may offer further insights and enhance the generalizability of our findings.

## Conclusion

Our finding offers several notable advantages in the field of sleep arousal research. First and foremost, it proposed an automatic scoring method for arousal intensity, which does not require relying on human experts to inspect all EEG recordings and subjectively score them visually. Additionally, our proposed methodology introduces a refined and more granular approach to assessing sleep arousal intensity by analyzing it by each of the four sleep stages, contributing to a more nuanced understanding of sleep fragmentation. By categorizing arousal intensity into five distinct levels and employing machine learning techniques, this method enhances the precision and objectivity of sleep quality assessment. In conclusion, this study successfully developed classifiers optimized for each sleep stage to improve the accuracy of arousal intensity classification using machine learning techniques. The optimized classifiers showed high sensitivity and specificity for each sleep stage, achieving an average sensitivity of 82.68%, specificity of 95.68%, and AUROC of 96.30%. The sensitivity of the control, arousal intensity level 0, was 83.07%, a 1.25% increase over the unoptimized model. The findings shed light on the unique characteristics of arousal intensity during different sleep stages, and the selected features and classifiers with the highest accuracy demonstrated common characteristics. Developing a classifier for each sleep stage based on the concept of arousal intensity represents a novel approach to sleep arousal research. It has important implications for the future development of more accurate and effective predictive models in sleep research. The classification model, which is optimized for each sleep stage, not only provides a reliable means of distinguishing between different arousal intensity levels but also offers a valuable tool for researchers and clinicians seeking to quantify sleep disturbances accurately. Furthermore, the novel approach of this study to arousal research holds the promise of advancing the development of more accurate and helpful models in the field of sleep research, ultimately benefiting individuals affected by sleep disorders and promoting improved overall health.

## Data Availability

The data that support the findings of this study are available from Samsung Medical Center, but restrictions apply to the availability of these data, which were used under license for the current study, and so are not publicly available. Data are available from the authors upon reasonable request and with permission of Samsung Medical Center (eunyeon.joo@gmail.com).
